# Upper limb motor recovery using a digital health–driven IoT rehabilitation system: a case report

**DOI:** 10.3389/fresc.2026.1767786

**Published:** 2026-04-13

**Authors:** Shuichi Sasaki, Naonobu Takahira, Natsuki Sato, Takayuki Watabe, Michiteru Miyazaki, Syuhei Chiba, Sora Kurosaki, Yutaka Ishii, Takuya Nakai, Atsuko Karube, Akari Kobayashi, Kouji Tsuda

**Affiliations:** 1 Department of Rehabilitation, Kitasato University Hospital, Sagamihara, Japan; 2 School of Allied Health Sciences, Kitasato University, Sagamihara, Japan; 3 Rehabilitation Center, Showa University Fujigaoka Rehabilitation Hospital, Yokohama, Japan; 4 Faculty of Medicine, Osaka Medical and Pharmaceutical University, Takatsuki, Japan

**Keywords:** case report, closed-loop system, digital health, IoT rehabilitation, motor learning, real-time feedback, stroke rehabilitation, upper limb recovery

## Abstract

**Background:**

Digital health–driven rehabilitation systems incorporating Internet of Things (IoT) technologies have attracted increasing attention as a means to support upper limb motor recovery after stroke. However, detailed clinical descriptions of their implementation in routine inpatient rehabilitation remain limited.

**Case presentation:**

We report the case of a right-handed man in his forties with right-sided upper limb motor impairment following putaminal hemorrhage. The patient underwent rehabilitation using a digital health–driven IoT-based upper limb rehabilitation system starting approximately one month after stroke onset during the convalescent rehabilitation phase.

**Intervention:**

The intervention was conducted over a two-week period, consisting of 10 sessions (approximately 40 min per session) as part of routine inpatient rehabilitation. The system integrated a portable smart projector, a three-dimensional motion capture sensor, and a communication robot to deliver interactive, task-oriented training. Five activities of daily living–oriented tasks (wiping, unlocking, squeezing, cup transfer, and typing) were implemented, with task difficulty adjusted by the treating occupational therapist according to the patient's performance.

**Outcomes:**

Upper limb motor function assessed by the Fugl–Meyer Assessment for the Upper Extremity improved from 63 to 66. Real-world arm use assessed by the Motor Activity Log showed an Amount of Use score of 5 both before and after the intervention, suggesting a ceiling effect, while the Quality of Movement score improved slightly from 4.8 to 5. The patient demonstrated high engagement and adherence throughout the intervention, and no adverse events were observed.

**Conclusion:**

This case report demonstrates the clinical feasibility of integrating a digital health–driven IoT rehabilitation system into routine inpatient stroke rehabilitation. Although generalization is limited by the single-case design, the present case highlights the potential of IoT-based digital health technologies to support task-oriented training and patient engagement in upper limb rehabilitation.

## Introduction

With the increasing integration of digital health (DH) technologies in rehabilitation, Internet of Things (IoT) solutions offer new pathways for patient-centered care.

In recent years, the importance of rehabilitation for patients with upper limb motor paralysis following cerebrovascular disease has been increasingly recognized. According to the World Health Organization (WHO) and major epidemiological studies, stroke is a leading neurological disorder worldwide, affecting approximately 12 million new individuals annually (1). Among these, upper limb motor paralysis occurs in about 70%–80% of patients, resulting in significant impairments in the performance of activities of daily living (ADLs), such as eating, grooming, and dressing, and creating a strong demand for rehabilitative interventions (2).

For these patients, the ultimate goal of rehabilitation is to achieve functional recovery that enables independent and effective use of the affected limb in daily life. A representative intervention method is Constraint-Induced Movement Therapy (CIMT), which promotes neural plasticity and functional generalization to ADL contexts by restricting use of the non-paretic limb and compelling active use of the paretic upper limb (3).

Moreover, to generalize upper limb function to ADL tasks, it is not sufficient to merely improve muscle strength or joint range of motion. Repetitive, task-specific training in environments that closely simulate real-life situations is essential (4). Therefore, from the early stages of rehabilitation, clinicians must ensure that patients are consciously aware of how the affected upper limb can be adapted to ADL tasks. However, this awareness can be influenced by patients' psychological states, cognitive capacities, and the limitations of the rehabilitation environment.

To address these challenges, technological approaches combining robotics and IoT technologies have recently gained attention. These systems enable intuitive and repetitive intention-driven motor training and are reported to contribute to sustained patient motivation (5).

Furthermore, integrating IoT with robotic assistance makes it possible to implement ADL-oriented task-specific training, which has been shown to enhance patients' self-efficacy and satisfaction with rehabilitation (6). The inclusion of sensors and app-based feedback functions further facilitates interactive communication between healthcare providers and patients, establishing a novel form of remote rehabilitation (7).

In addition, a recent systematic review and meta-analysis reported that technology-assisted and home-based upper limb rehabilitation after stroke was associated with significant improvements in motor function and activities of daily living outcomes, supporting the growing role of digital and sensor-based systems in post-stroke care (8). Moreover, a recent scoping review synthesizing a broad range of studies reported that home-based digital technologies—such as sensors, virtual reality, mobile applications, and telerehabilitation platforms—have been increasingly adopted for post-stroke upper limb rehabilitation, demonstrating expanding feasibility and clinical applicability of technology-assisted programs in real-world settings (9). In addition, a recent Cochrane systematic review reported that virtual reality–based interventions could improve upper limb function after stroke compared with conventional therapy, highlighting the clinical relevance of technology-assisted rehabilitation approaches (10). Furthermore, a recent systematic review focusing on Internet of Things (IoT)–enabled rehabilitation systems reported that connected sensors, wearable devices, and networked platforms facilitate continuous monitoring, data-driven feedback, and personalized interventions in stroke rehabilitation, thereby enhancing clinical feasibility and scalability of digital health solutions (11). Additionally, a systematic review of telerehabilitation approaches reported that remote, technology-mediated interventions can yield meaningful improvements in upper limb function after stroke, supporting the practicality of networked rehabilitation models for continuous care beyond the clinic (12).

Against this background, this case report describes the clinical application of a digital health–driven IoT rehabilitation system for upper limb motor recovery in a patient with stroke.

## Case presentation

### Patient information

The patient was a right-handed man in his forties who developed unilateral upper limb motor impairment following putaminal hemorrhage. After completion of acute medical treatment, he was admitted to a convalescent rehabilitation ward for intensive rehabilitation aimed at improving activities of daily living (ADLs). The intervention using the digital health–driven IoT rehabilitation system was initiated about one month after stroke onset. Written informed consent for participation and publication was obtained from the patient prior to the intervention, and the study protocol was approved by the institutional review board.

The patient had no severe cognitive impairment or aphasia that would interfere with task comprehension or interaction with the digital rehabilitation system. No major orthopedic or neurological comorbidities affecting upper limb function were present.

**Figure 1:**
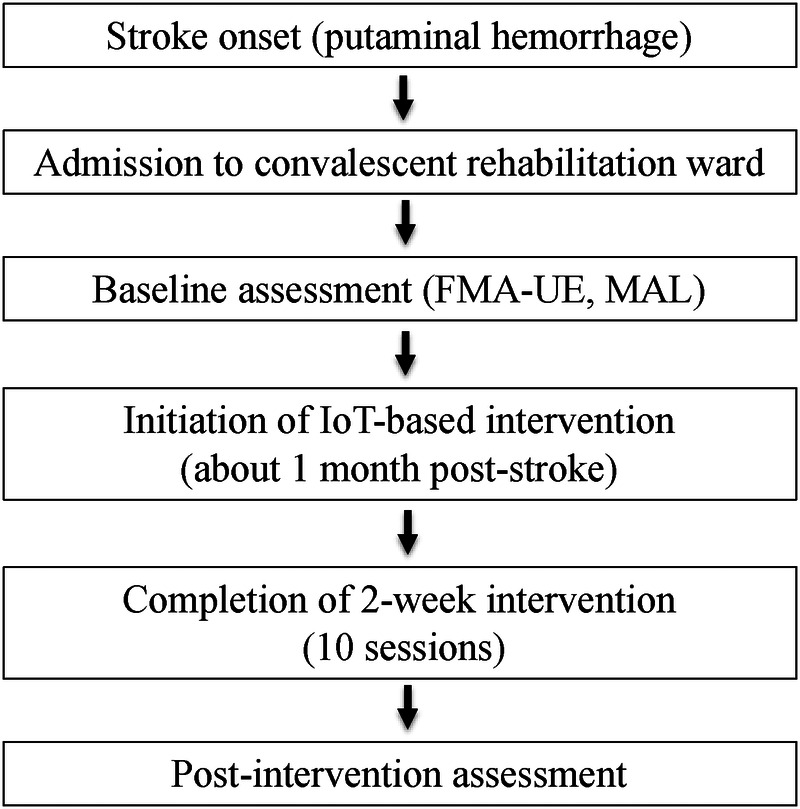
Clinical timeline of the case. The figure illustrates the clinical course from stroke onset to post-intervention assessment, including admission to the convalescent rehabilitation ward, baseline evaluation, initiation of the IoT-based intervention approximately one month after stroke onset, completion of the two-week intervention, and outcome assessment.

A clinical timeline summarizing the patient's course from stroke onset to post-intervention assessment is illustrated in Figure 1.

### Clinical findings and baseline assessment

At baseline, the patient exhibited impaired voluntary motor control of the affected upper limb, which limited spontaneous use during daily activities. Upper limb motor function was assessed using the Fugl–Meyer Assessment for the Upper Extremity (FMA-UE). Functional independence was evaluated using the Functional Independence Measure (FIM), and real-world arm use was assessed with the Motor Activity Log (MAL), including the Amount of Use (AOU) and Quality of Movement (QOM) subscales.

At baseline, the FIM score was 119 out of 126, indicating a high level of overall independence. The FMA-UE score was 63, reflecting moderate motor impairment of the affected upper limb. The MAL-AOU score was 5, suggesting frequent use of the affected limb in daily life, while the MAL-QOM score was 4.8, indicating slight impairment in movement quality. Based on these findings, a task-oriented rehabilitation approach incorporating real-time feedback was selected to facilitate motor relearning and functional use of the affected upper limb.

### Timeline

A clinical timeline summarizing the patient's course from stroke onset to post-intervention assessment is presented in Figure 1. The timeline includes stroke onset, admission to the convalescent rehabilitation ward, baseline evaluation, initiation of the IoT-based intervention, completion of the intervention, and outcome assessment.

### Intervention and system overview

The patient participated in a two-week rehabilitation program using a digital health–driven IoT-based upper limb rehabilitation system as part of routine inpatient rehabilitation. The intervention consisted of 10 sessions (five sessions per week), with each session lasting approximately 40 min.

**Figure 2:**
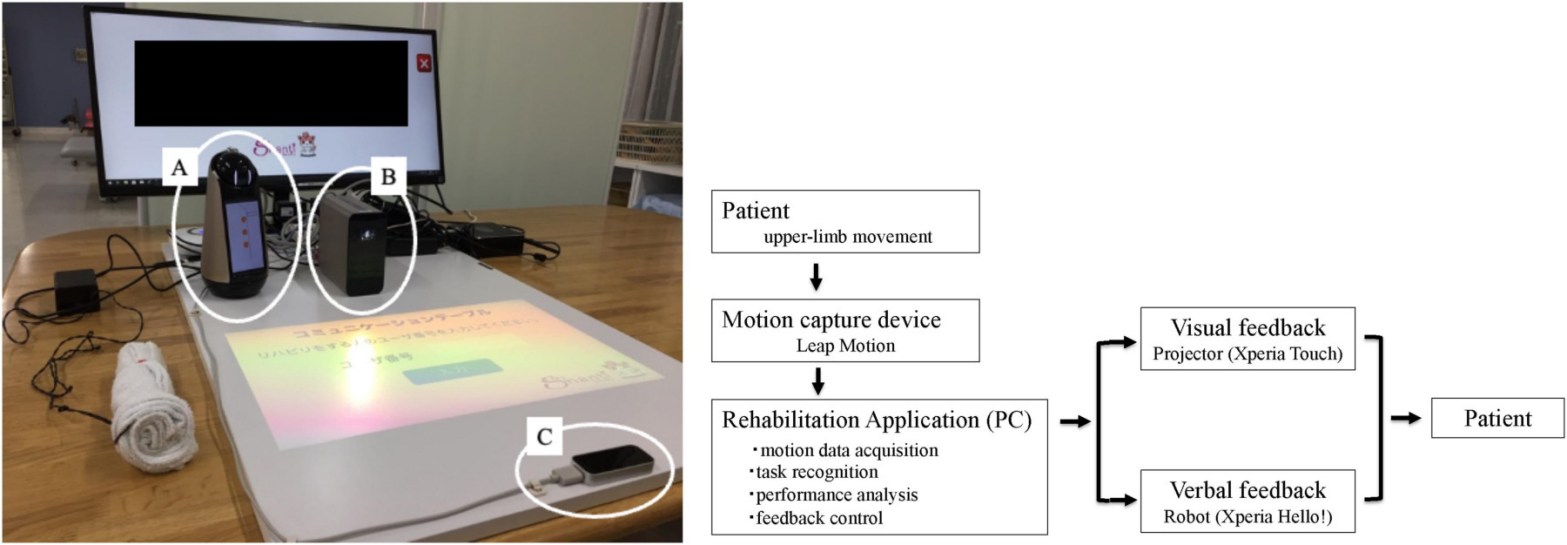
IoT-based rehabilitation system architecture and data flow. The left panel shows the physical configuration of the system, including a communication robot **(A)**, a portable smart projector **(B)**, and a three-dimensional motion capture device **(C)**. The right panel illustrates the system architecture and data flow of the IoT-based rehabilitation system. Upper-limb movements performed by the patient are captured in real time by the motion sensor and transmitted to a personal computer. Within the computer, the rehabilitation application processes the data through four functional modules: motion data acquisition, task recognition, performance analysis, and feedback control. These modules operate sequentially to transform raw motion data into performance-based feedback signals. Based on this processing, multimodal feedback is generated and delivered to the patient via visual projection and verbal interaction. The feedback is continuously adjusted according to the patient's performance, thereby forming a real-time closed-loop system that enables interactive and adaptive rehabilitation.

The system integrated a portable smart projector (Xperia Touch, Sony), a three-dimensional motion capture sensor (Leap Motion Controller, Ultraleap), a communication robot (Xperia Hello!, Sony), and a base station (Raspberry Pi, RS Components). This configuration enabled interactive, task-oriented upper limb training with real-time motion sensing, visual feedback, and verbal encouragement. The overall system configuration is illustrated in Figure 2. During each session, the patient performed task-oriented upper limb exercises using the IoT-based system under therapist supervision. The therapist selected and adjusted tasks based on the patient's motor performance, ensuring an appropriate level of challenge.

Real-time visual and verbal feedback were provided continuously during task execution, allowing the patient to modify movements based on performance. This adaptive feedback mechanism was intended to facilitate motor learning and maintain engagement throughout repetitive training.

### IoT system architecture

The rehabilitation system was designed as a closed-loop IoT-based architecture integrating motion capture, real-time data processing, and multimodal feedback. Upper-limb movements were captured using a three-dimensional motion sensor (Leap Motion, Ultraleap Ltd., UK) and transmitted to a personal computer in real time. The rehabilitation application processed the data through four functional modules: motion data acquisition, task recognition, performance analysis, and feedback control.

The motion data acquisition module collected kinematic information from the sensor. The task recognition module identified the performed movement based on predefined task parameters. The performance analysis module quantified task execution, including movement accuracy and completion. The feedback control module generated adaptive feedback signals according to the patient's performance.

The processed information was delivered to the patient via visual projection and verbal interaction. This continuous interaction between patient performance and system response constituted a real-time closed-loop system, enabling interactive and adaptive motor learning.

### Task design and interactive features

**Figure 3:**
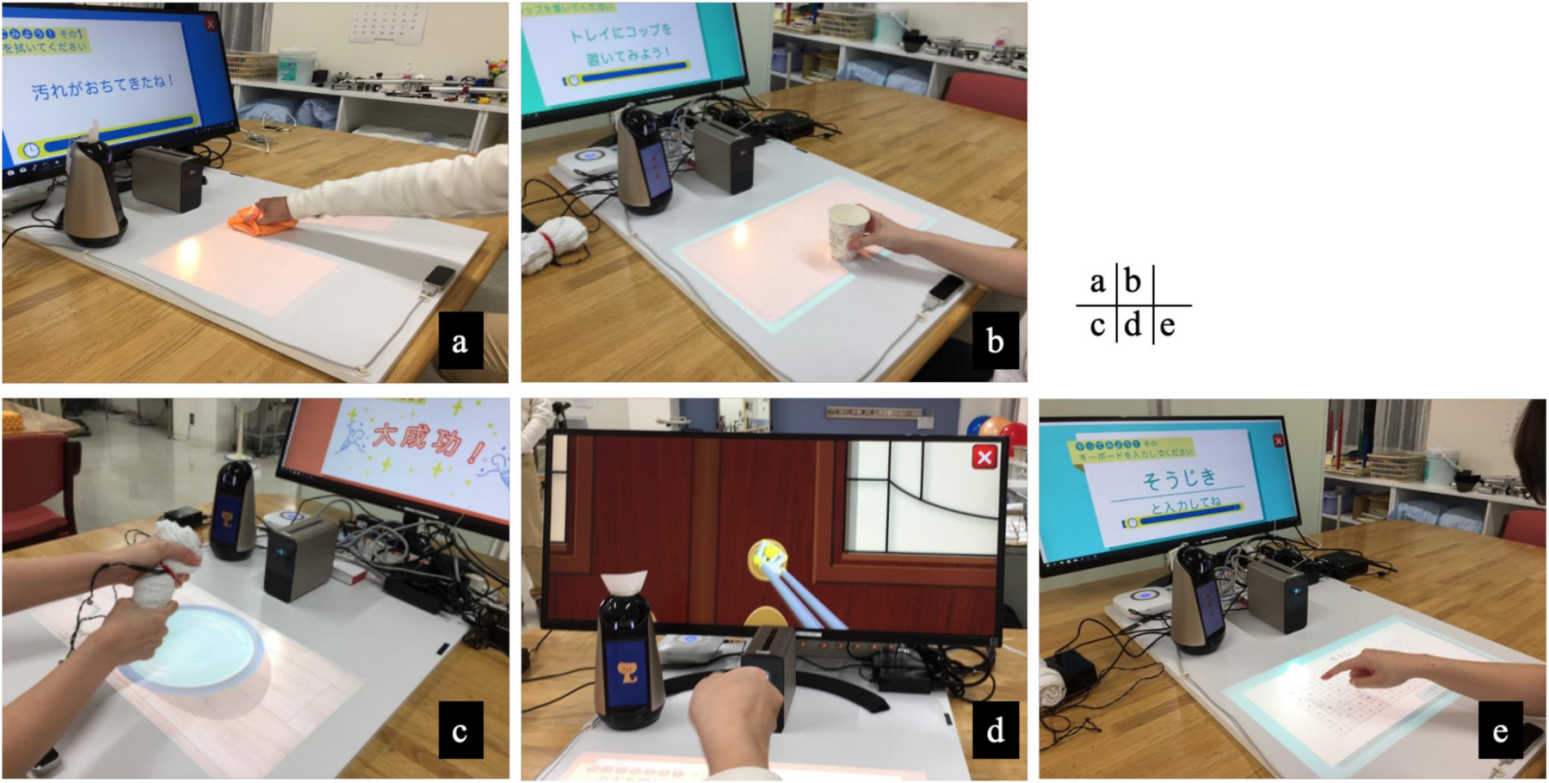
Task content of the IoT-based rehabilitation application. Five task-based exercises were implemented to promote diverse upper limb movements. Each panel shows one of the interactive tasks: **(a)** Wiping task: The user wipes a virtual dirty surface, promoting shoulder and elbow extension. **(b)** Unlocking task: The user grasps and turns a projected key to unlock a door, training wrist rotation and grip. **(c)** Squeezing task: The user squeezes a pressure-sensitive towel, visualized as water drops into a bowl, encouraging grip strength and forearm activity. **(d)** Cup transfer task: The user picks up and moves a virtual cup to a target area, supporting coordination and object manipulation. **(e)** Typing task: The user types on a virtual keyboard projected onto the tabletop, improving fine motor control and finger dexterity.

Five ADL-related tasks—wiping, unlocking, squeezing, cup transfer, and typing—were implemented to promote reaching, grasping, forearm rotation, object manipulation, and fine motor control. These tasks were designed to be intuitive, adaptable in difficulty, and closely aligned with real-life upper limb functions. Task selection and difficulty were adjusted by the treating occupational therapist based on the patient's motor performance and engagement. An overview of the task content is shown in Figure 3.

The system incorporated multiple interactive features to enhance user engagement and motor learning. Task difficulty levels were adjustable, and the communication robot provided real-time verbal encouragement. Visual and auditory feedback were integrated to reinforce movement execution, such as dynamic visualization of grip force during the squeezing task and motion-dependent task progression during the unlocking task. These multisensory, goal-oriented interactions were intended to support motivation and sustained participation during repetitive training.

### Intervention protocol and outcomes

All sessions were conducted in a hospital-based rehabilitation room under the supervision of licensed rehabilitation therapists to ensure safety and appropriate task execution. No changes were made to concomitant standard rehabilitation programs during the intervention period.

After completion of the two-week intervention, upper limb motor function assessed by the Fugl–Meyer Assessment for the Upper Extremity (FMA-UE) increased from 63 to 66. Real-world arm use assessed by the Motor Activity Log (MAL) showed an Amount of Use (AOU) score of 5 both before and after the intervention, suggesting a ceiling effect, while the Quality of Movement (QOM) score improved slightly from 4.8 to 5. The patient demonstrated high levels of engagement and adherence throughout the intervention, and no adverse events were observed.

## Discussion

The purpose of this case report was to describe the clinical application and feasibility of a digital health–driven IoT rehabilitation system for upper limb motor recovery in a patient with stroke. This purpose was achieved by demonstrating how an ADL-oriented, task-specific digital system can be integrated into routine inpatient rehabilitation and adapted by therapists according to patient performance.

The scientific contribution of this report lies not in establishing clinical efficacy, but in providing a detailed clinical description of system implementation. Specifically, this case illustrates how IoT-based motion sensing, interactive visual feedback, and therapist-guided task adaptation can be combined within a single platform to support patient engagement and functional use of the affected upper limb in a real-world rehabilitation setting. By focusing on feasibility and clinical integration, this report complements existing efficacy-focused studies and contributes practical knowledge for the translation of digital health technologies into everyday rehabilitation practice.

Although only modest changes were observed in impairment- and activity-level outcomes over the short intervention period, the results are clinically plausible given the relatively high baseline functional status and the limited duration of the intervention. Notably, the MAL-AOU score was already at the maximum level at baseline, suggesting a ceiling effect that may have limited the ability to detect further changes in the frequency of arm use. In contrast, the slight improvement observed in MAL-QOM suggests potential qualitative benefits in movement execution during daily activities.

In the context of expanding digital health ecosystems, this case illustrates how smart devices and user-centered interfaces may be harnessed to address post-stroke motor impairments. Recent studies have highlighted the importance of real-time feedback and technology-assisted training in promoting motor recovery after stroke (13, 14). Sensor-based rehabilitation systems have been shown to facilitate continuous performance monitoring and adaptive intervention, supporting more effective motor learning processes (15). One of the core features of the present IoT-based rehabilitation system is its ability to simulate ADL-like tasks through interactive visual projections and real-time feedback. Tasks such as wiping, unlocking, cup transfer, and typing targeted various upper limb movement components—including reaching, grasping, forearm rotation, and finger individuation—that are directly relevant to daily activities. The use of multisensory feedback and verbal encouragement provided by the communication robot may have contributed to enhanced motivation and engagement during repetitive motor training, consistent with established principles of motor learning and behavioral reinforcement.

The present system differs from conventional rehabilitation approaches in that it integrates motion capture, real-time data processing, and adaptive feedback within a closed-loop IoT architecture. This enables continuous interaction between patient performance and system response in real time, which may enhance motor learning compared to conventional therapist-dependent feedback.

Furthermore, the modular design of the system allows for scalable and flexible implementation in clinical settings, supporting the integration of digital health technologies into routine rehabilitation practice.

The system also allowed for adjustable task difficulty and interface customization, enabling individualized adaptation according to the patient's motor performance. Such flexibility is particularly important in stroke rehabilitation, where motor impairments and cognitive capacities vary widely across individuals. Tailoring task demands to the patient's ability may help maintain engagement while avoiding excessive physical or cognitive burden.

From a theoretical perspective, this case supports the potential integration of a behaviorally grounded approach—similar to the “transfer package” used in Constraint-Induced Movement Therapy—into technology-assisted rehabilitation. The transfer package has been shown to play a crucial role in facilitating the generalization of motor gains from the clinical setting to real-world use of the paretic limb in activities of daily living (16–19). By embedding task-oriented training within an engaging and interactive digital environment, the present system may offer a practical platform for supporting such transfer processes.

Taken together, this case does not aim to establish the efficacy of the intervention, but rather provides a detailed clinical example of how an IoT-based digital health system can be implemented in routine inpatient rehabilitation. This case highlights the potential of IoT-based rehabilitation systems to bridge engineering design and clinical application, supporting the future development of scalable and adaptive rehabilitation strategies.

## Limitations

Several limitations of this case report should be acknowledged. First, this study is based on a single patient, which precludes generalization of the findings. Second, the intervention period was relatively short, and long-term effects on upper limb function and real-world arm use could not be evaluated. Third, although standardized outcome measures were used, some outcomes—such as the Motor Activity Log Amount of Use—were subject to a ceiling effect due to the patient's high baseline functional level. Finally, causal relationships between the intervention and observed changes cannot be established, as spontaneous recovery and contextual factors may have influenced the results. Future studies involving larger patient cohorts, broader baseline severity, and longer follow-up periods are warranted to clarify the clinical effectiveness of IoT-based digital health rehabilitation systems.

## Conclusion

This case report describes the clinical application of a digital health–driven IoT rehabilitation system for upper limb motor recovery after stroke. The present case suggests that integrating task-oriented training with interactive digital feedback may support patient engagement and qualitative aspects of upper limb use in daily activities. Although generalization is limited, this report highlights the potential clinical utility of IoT-based digital health technologies as a feasible and adaptable tool within routine upper limb rehabilitation practice.

## Data Availability

The original contributions presented in the study are included in the article/Supplementary Material, further inquiries can be directed to the corresponding author.
